# Supplementary material for: Predictors of frequency of CF care in the US Cystic Fibrosis Foundation Patient Registry
Source: PLoS One. 2024 Dec 3;19(12):e0313510. doi: 10.1371/journal.pone.0313510 (PMC11614261; doi:10.1371/journal.pone.0313510)

S1 Fig. A directed acyclic graph (DAG) that illustrates the hypothesized causal relationships among the covariates included in our statistical models. The DAG guided the selection and adjustment of variables in our analyses to account for potential confounding pathways. This version of the DAG is color coded to represent one of our models, the analysis of the relationship between underweight BMI and between visit interval (BVI). White circles are variables that comprise the minimally sufficient adjustment set and are adjusted for in our model. Red denotes an ancestor of exposure *and* outcome, while blue denotes an ancestor of outcome only. Black lines denote causal relationships between variables, and green lines represent the causal relationship of interest (direct and indirect pathways). Figure created using DAGitty (http://dagitty.net/).

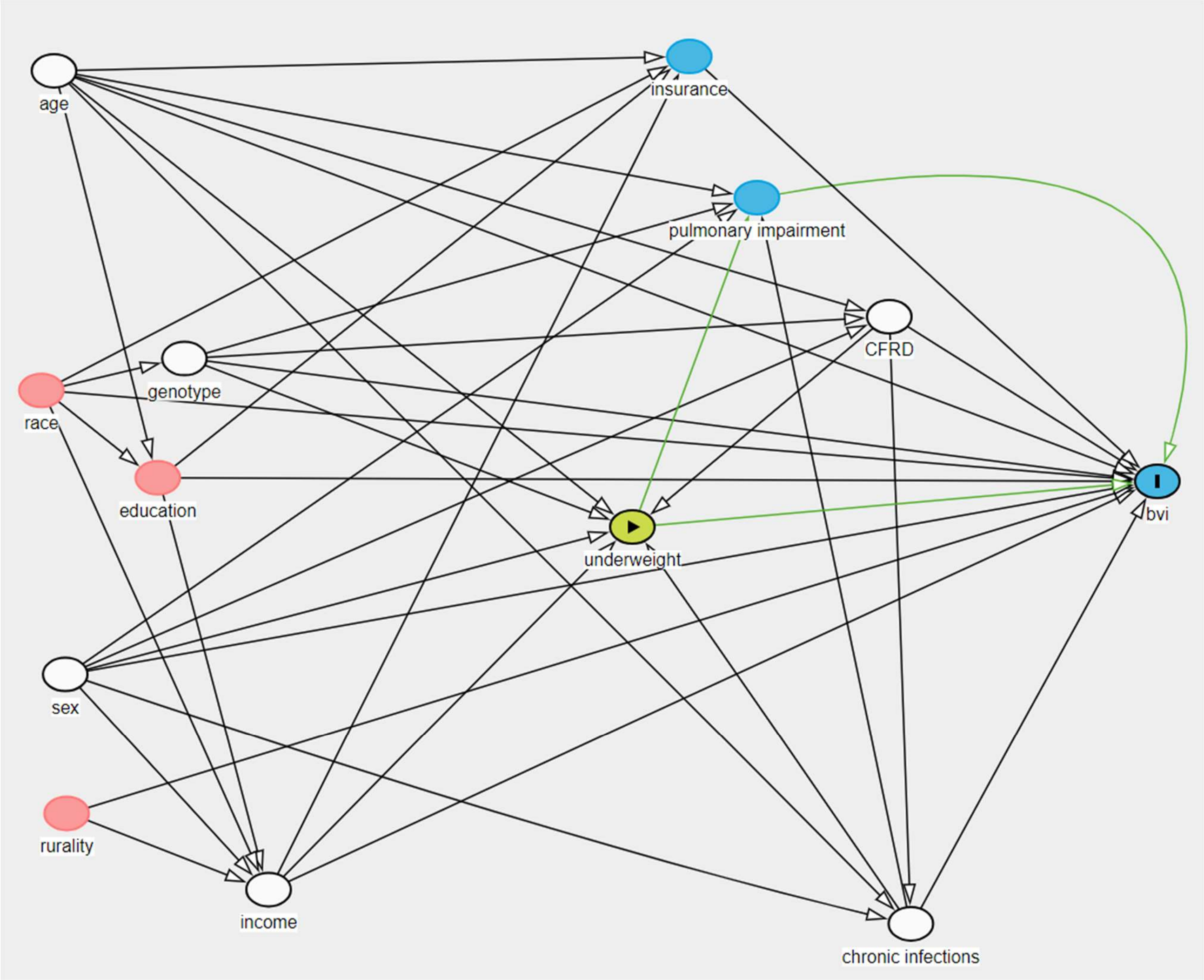

Supplement: S1 Fig — The DAG guided the selection and adjustment of variables in our analyses to account for potential confounding pathways. This version of the DAG is color coded to represent one of our models, the analysis of the relationship between underweight BMI and between visit interval (BVI). White circles are variables that comprise the minimally sufficient adjustment set and are adjusted for in our model. Red denotes an ancestor of exposure and outcome, while blue denotes an ancestor of outcome only. Black lines denote causal relationships between variables, and green lines represent the causal relationship of interest (direct and indirect pathways). Figure created using DAGitty (http://dagitty.net/). (PDF) [file pone.0313510.s001.pdf]
