# Supplementary material for: Predictors of frequency of CF care in the US Cystic Fibrosis Foundation Patient Registry
Source: PLoS One. 2024 Dec 3;19(12):e0313510. doi: 10.1371/journal.pone.0313510 (PMC11614261; doi:10.1371/journal.pone.0313510)

S2 Fig. Findings from an ad hoc analysis evaluating changes in BVI in those with at least one G551D mutation before and after 2012 FDA approval of ivacaftor. Panel **a.)** shows the number of individuals with the G551D mutation by age, before (light green) and after 2012 (dark green), **b.)** shows estimated between-visit interval across the lifespan before and after 2012 FDA approval of ivacaftor. Color shaded areas indicate 95% confidence bounds. The gray shaded area indicates the current CF Foundation-recommended between-visit interval of 90 days for reference.

a.

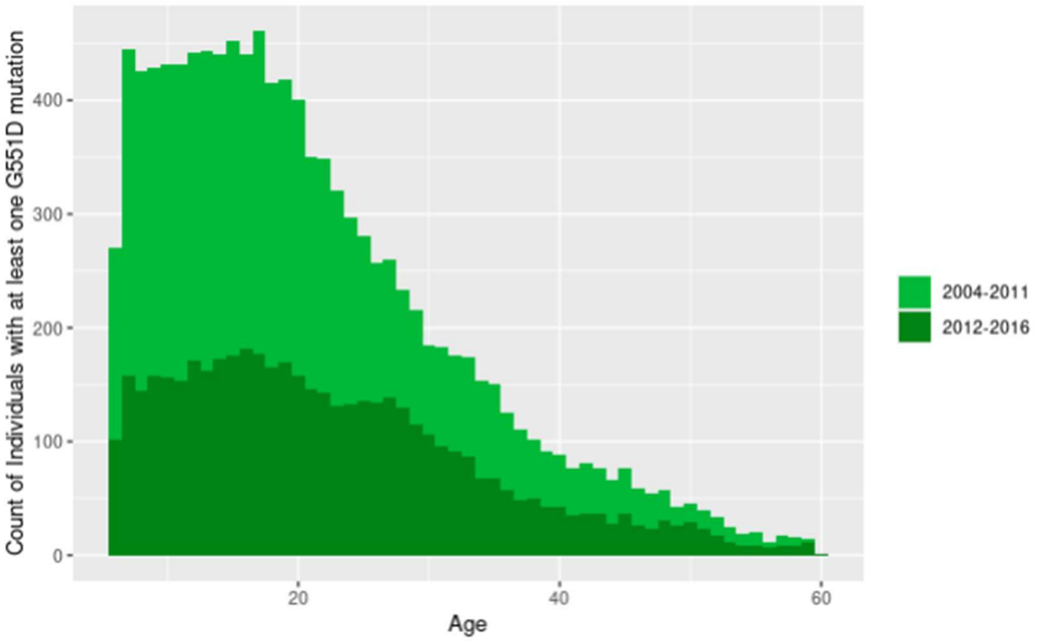

b.

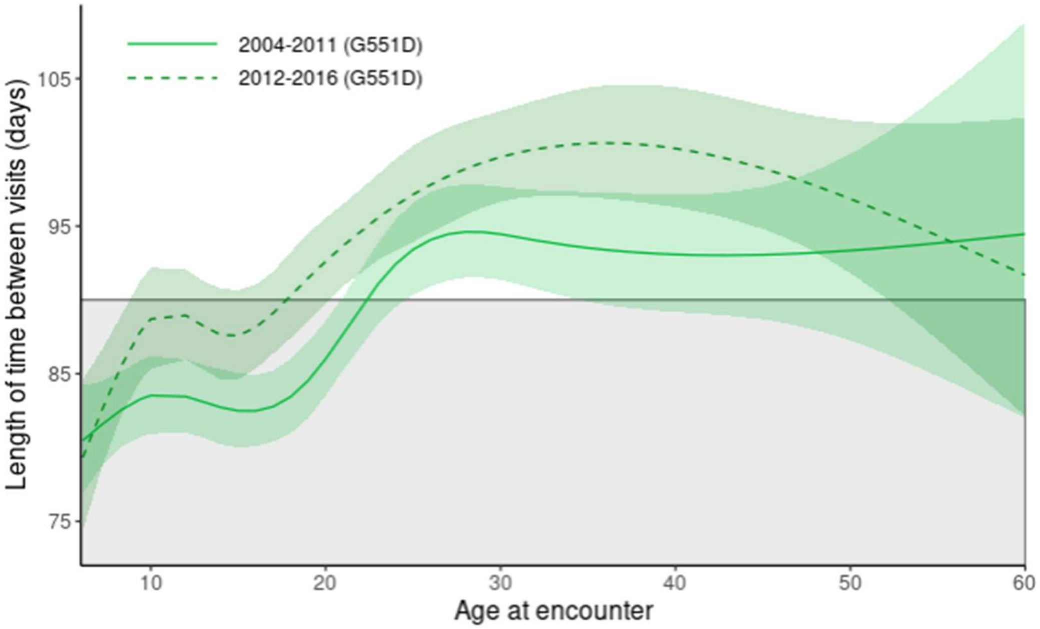

Supplement: S2 Fig — Panel a.) shows the number of individuals with the G551D mutation by age, before (light green) and after 2012 (dark green), b.) shows estimated between-visit interval across the lifespan before and after 2012 FDA approval of ivacaftor. Color shaded areas indicate 95% confidence bounds. The gray shaded area indicates the current CF Foundation-recommended between-visit interval of 90 days for reference. (PDF) [file pone.0313510.s002.pdf]
