# Supplementary material for: Predictors of frequency of CF care in the US Cystic Fibrosis Foundation Patient Registry
Source: PLoS One. 2024 Dec 3;19(12):e0313510. doi: 10.1371/journal.pone.0313510 (PMC11614261; doi:10.1371/journal.pone.0313510)
Supplement: S1 Table — (PDF) [file pone.0313510.s003.pdf]

S1 Table. Study variable definitions.

Variable Description	Variable name	Definition
Between Visit Interval (outcome)	BVI	BVI was calculated for each encounter as days from the prior encounter. Visits with BVI less than 30 days were excluded in the primary analysis, as the data did not specify inpatient or outpatient settings, and such short intervals typically indicate acute issue management rather than ongoing care. BVI was log-transformed the outcome to address skewed data distributions and mitigate the influence of outliers.
**Sociodemographic Factors**		
Patient identifier	Patient_ID	Unique encrypted patient ID supplied by the USCFFPR
Center identifier	CF_Clinic_ID	Unique encrypted clinic ID supplied by the USCFFPR
Age	encounterage	Patient age at clinical encounter as provided by the USCFFPR, rounded to two decimal points. Where patients had multiple encounters with the same age, an additional decimal point was assigned (for example, if there were three visits for a patient at age 18.50, the ages would be modified to 18.501, 18.502, and 18.503).
Sex	sex	Male and female, as documented in the USCFFPR
Race/Ethnicity	non_white	Categories include White and Non-white (Hispanic, Black or African American, American Indian or Alaska Native, Asian, Native Hawaiian or Other Pacific Islander, and Other), as documented in the USCFFPR. Multiple races and ethnicities could be selected. Records with selections other than only White and non-Hispanic were grouped together as Non-white. Value of 1 assigned if race or ethnicity as endorsed, 0 if not endorsed or missing.
Rurality	rurality	Rurality was defined by applying 2010 RUCA Codes to residential zip codes. We used Categorization A as defined by The University of Washington School of Medicine's WWAMI Rural Health Research Center.[1] Time-varying, value from prior visit. Originally missing from 8% of encounters, 91% of missing values were imputed using LOCF/NOCB.
Insurance type	insurance_type	Patients may have reported having more than one insurance provider. For this study, individuals were sorted into only one category of insurance coverage using the following hierarchy: (1) Private insurance/military/parent's insurance vs (2) Medicare, Medicaid, State, Indian Health Service vs (3) other vs (4) Patient had no health insurance for the entire year, as documented in the USCFFPR. Time-varying, value from prior visit.
Family income	family_income	Family income was grouped in the following categories for analysis: <$40,000, $40,000 to $90,000,>$90,000. Time-varying, value from prior visit. Originally missing from 77% of encounters, 48% of missing values were imputed using LOCF/NOCB.
Education	highest_ed	Defined as the highest education in the family (maximum of pwCF, spouse, mother, father) at each encounter. Time-varying, value from prior visit. Originally missing from 3% of encounters, 0% of missing values were imputed using LOCF/NOCB.
Education (grouped)	college_ed	Defined using highest_ed to create a two-level variable; 1) College Degree (College Graduate, Masters/Doctoral level degree) versus 2) No College Degree (Less than High School, High School diploma or equivalent, Some College).

Race/Ethnicity and Insurance	race_insurance	A six-level categorical variable with values of “Non-White - Private", "Non-White - Public", "Non-White - Other", "White - Private", "White - Public", "White - Other". Race/ethnicity and insurance are defined as described above, except the “Other” category includes other insurance, no insurance, and unknown insurance status.
**Disease-related factors**		
Genotype	genotype	F508del homozygote, heterozygote, and other/unknown as documented in the USCFFPR.
Underweight	underweight_BMI	Value of 1 assigned if BMI< 18.5 (for adults) or BMI Percentile < 5% (for pediatric patients), else 0. Time-varying, value from prior visit. Originally missing from 5% of encounters, 100% of missing values were imputed using LOCF/NOCB.
CF Related Diabetes	diabetes	CFRD documented annually in registry with or without fasting hyperglycemia (2-h PG >= 200). Value of 0 assigned prior to diagnosis, 1 upon diagnosis and all subsequent visits. Time-varying, value from prior visit.
Pseudomonas aeruginosa infection	chronic_pseudomonas	Chronically infected if either mucoid phenotype or 3 positive cultures during the study period. Patients considered infected from date of first culture. Time-varying, value from prior visit.
MRSA infection	chronic_MRSA	Chronically infected if had 3 positive cultures during the study period; patients were considered infected as of the date of the first culture. Time-varying, value from prior visit.
Burkholderia spp. infection	chronic_burkholderia	Chronically infected if had 2 positive cultures with any *Burkholderia* subtype during the study period; patients were considered infected as of the date of the first culture. Time-varying, value from prior visit.
Number of chronic infections	num_infections	Number of chronic infections, which can include p*seudomonas aeruginosa,* MRSA, and *burkholderia* spp., as defined above. Defined as no chronic infection if all three were not found to be present, 1 if only one of these three pathogens were found, 2 if two were found, and 3 if there was evidence of all three.
Pulmonary impairment	pulmonary_impairment	Categories of FEV1PP, standardized on GLI values per USCFFPR standard (continuous) as provided in the USCFFPR (variable name: GLI_FEV1_pct_predicted). Categories included mild (>=70%), moderate (41-69%), and severe (≤ 40%). Time-varying, value from prior visit. Originally missing from 12% of encounters, 100% of missing values were imputed using LOCF/NOCB.
Complications	complications	Values are 1 for all patients with severe lung impairment (FEV1PP ≤ 40) or underweight BMI or CF-related diabetes or chronically infected with Pseudomonas aeruginosa, MRSA, or Burkholderia spp, and 0 for those without these complications. Time-varying, value from prior visit.

Abbreviation list: BMI = Body mass index; CFRD = Cystic fibrosis-related diabetes; FEV1PP = Forced expiratory volume in one second; GLI = Global Lung Initiative; LOCF = Last observation carried forward; MRSA = Methicillin-resistant *Staphylococcus* aureus; NOCB = Next observation carried backward; RUCA = Rural-Urban Commuting Area; USCFFPR = United States Cystic Fibrosis Foundation Patient Registry; WWAMI = Washington, Wyoming, Alaska, Montana, Idaho

1. Rural Urban Commuting Area Codes Data. WWAMI Rural Health Research Center. Accessed July 1, 2024, https://depts.washington.edu/uwruca/ruca-uses.php
