# Supplementary material for: Predictors of frequency of CF care in the US Cystic Fibrosis Foundation Patient Registry
Source: PLoS One. 2024 Dec 3;19(12):e0313510. doi: 10.1371/journal.pone.0313510 (PMC11614261; doi:10.1371/journal.pone.0313510)
Supplement: S2 Table — Individual visits were excluded if pulmonary function testing (PFT) results or body mass index (BMI) was missing. Individuals were excluded if they had fewer than 2 visits between 2004–2016. (PDF) [file pone.0313510.s004.pdf]

S2 Table. Characteristics of people with cystic fibrosis age 6-60 years by inclusion status. Individual visits were excluded if pulmonary function testing (PFT) results or body mass index (BMI) was missing. Individuals were excluded if they had fewer than 2 visits between 2004-2016.

	**Excluded** N = 67 (0.2%)[1]	**Included** N = 28,588 (99.8%)[1]
***Sociodemographic* factors**		
**Age at first encounter**		
Median (IQR)	6 (6, 22)	12 (6, 21)
Range	6, 60	6, 60
**Age at CF Diagnosis, years**		
Median (IQR)	0.1 (0.0, 14.5)	0.5 (0.1, 3.3)
Range	-0.4, 60.8	-0.6, 60.7
**Birth Cohort**		
Before 1981	16 (24%)	5,629 (20%)
1981-1988	3 (4.5%)	5,558 (19%)
1989-1994	0 (0%)	5,376 (19%)
1995 and after	48 (72%)	12,025 (42%)
**Female**	35 (52%)	13,743 (48%)
**Race/Ethnicity**		
White	60 (90%)	26,926 (94%)
Hispanic	1 (1.5%)	2,011 (7.0%)
Black or African American	4 (6.0%)	1,256 (4.4%)
**Rurality** [2]		
Urban	50 (75%)	21,802 (76%)
Large rural	6 (9.0%)	3,210 (11%)
Small rural	1 (1.5%)	1,819 (6.4%)
Isolated	2 (3.0%)	1,524 (5.3%)
Missing/Unknown	8 (12%)	233 (0.8%)
**Insurance Coverage ***		
Private Insurance	26 (39%)	18,370 (64%)
Public Insurance	47 (70%)	23,141 (81%)
Other Insurance	2 (3.0%)	3,500 (12%)
Unknown Insurance	0 (0%)	494 (1.7%)
No Insurance	0 (0%)	1,572 (5.5%)
**Highest Education in Family** [3]		
Less than High School	2 (3.0%)	508 (1.8%)
High School diploma or equivalent	6 (9.0%)	3,694 (13%)
Some College	9 (13%)	4,769 (17%)
College Graduate	19 (28%)	12,362 (43%)
Masters/Doctoral level degree	9 (13%)	5,701 (20%)
Missing	22 (33%)	1,554 (5.4%)
**Maximum Family Income** [4]		

<$40,000	10 (15%)	4,777 (17%)
$40,000 to $90,000	10 (15%)	5,005 (18%)
>$90,000	8 (12%)	5,679 (20%)
Missing	39 (58%)	13,127 (46%)
Disease-related factors		
**Genotype**		
F508del heterozygote	28 (42%)	10,920 (38%)
F508del homozygote	24 (36%)	12,994 (45%)
Other/Unknown	15 (22%)	4,674 (16%)
**Pulmonary Impairment** [5]		
Mild (FEV1PP ≥ 70%)	3 (4.5%)	10,645 (37%)
Moderate (FEV1PP 41-69%)	13 (19%)	9,809 (34%)
Severe (FEV1PP <40%)	43 (64%)	8,134 (28%)
Unknown	8 (12%)	0 (0%)
**Underweight** *[6]	1 (1.5%)	9,505 (33%)
**Chronic Infection** *		
P. aeruginosa	5 (7.5%)	18,363 (64%)
MRSA	2 (3.0%)	9,590 (34%)
Burkholderia spp.	1 (1.5%)	1,606 (5.6%)
**CF-related Diabetes** *	5 (7.5%)	9,410 (33%)

[1] n (%), unless otherwise specified
[2] Most rural residential stratum during the study period
[3] Highest level of education among the individual, their parents, and/or spouse during the study period
[4] Highest reported income during the study period
[5] Lowest lung function stratum during study period
[6] BMI< 18.5 for adults or BMI Percentile < 5% for children
*Ever experienced during the study period. Total within the category may therefore exceed 100%.
MRSA = Methicillin-resistant *Staphylococcus aureus,* FEV1PP = Forced expiratory volume in one second, IQR = Interquartile range
