# Supplementary material for: Predictors of frequency of CF care in the US Cystic Fibrosis Foundation Patient Registry
Source: PLoS One. 2024 Dec 3;19(12):e0313510. doi: 10.1371/journal.pone.0313510 (PMC11614261; doi:10.1371/journal.pone.0313510)
Supplement: S3 Table — (PDF) [file pone.0313510.s005.pdf]

S3 Table. Length of between visit interval across study period.

Characteristic	**Overall**, N = 859,568[1]	**2004-2007**, N = 219,124[1]	**2008-2011**, N = 269,541[1]	**2012-2016**, N = 370,903[1]
Between Visit Interval				
<= 3m (<= 90 days)	471,657 (55%)	120,760 (55%)	148,222 (55%)	202,675 (55%)
3-4m (91-120 days)	236,588 (28%)	59,169 (27%)	72,725 (27%)	104,694 (28%)
4-5m (121-150 days)	57,351 (6.7%)	14,960 (6.8%)	17,952 (6.7%)	24,439 (6.6%)
5-6m (151-180 days)	29,196 (3.4%)	7,726 (3.5%)	9,242 (3.4%)	12,228 (3.3%)
6-9m (151-270 days)	38,437 (4.5%)	10,447 (4.8%)	12,121 (4.5%)	15,869 (4.3%)
9m-1y (271-365 days)	12,392 (1.4%)	3,272 (1.5%)	4,150 (1.5%)	4,970 (1.3%)
1y+ (366+ days)	13,947 (1.6%)	2,790 (1.3%)	5,129 (1.9%)	6,028 (1.6%)

[1] n (%)

Note: individuals may contribute data to more than one time period
