# Supplementary material for: Predictors of frequency of CF care in the US Cystic Fibrosis Foundation Patient Registry
Source: PLoS One. 2024 Dec 3;19(12):e0313510. doi: 10.1371/journal.pone.0313510 (PMC11614261; doi:10.1371/journal.pone.0313510)
Supplement: S4 Table — (PDF) [file pone.0313510.s006.pdf]

S4 Table. Frequency of prolonged gaps by age group.

Characteristic	Overall<br>N = 28,588[1]	Pediatric<br>N = 18,924[1]	Adult<br>N = 19,096[1]
6 month gap	18,047 (63%)	7,949 (42%)	12,842 (67%)
12 month gap	8,404 (29%)	2,267 (12%)	6,570 (34%)
18 month gap	4,584 (16%)	944 (5.0%)	3,727 (20%)

[1] n (%)

Note: individuals may contribute data to pediatric and adult statistics
