# Supplementary material for: Predictors of frequency of CF care in the US Cystic Fibrosis Foundation Patient Registry
Source: PLoS One. 2024 Dec 3;19(12):e0313510. doi: 10.1371/journal.pone.0313510 (PMC11614261; doi:10.1371/journal.pone.0313510)
Supplement: S5 Table — (PDF) [file pone.0313510.s007.pdf]

S5 Table. Frequency of prolonged gaps by pediatric age brackets.

Characteristic	**Age 6-8**, N = 9,818[1]	**Age 8-10**, N = 10,889[1]	**Age 10-12**, N = 11,190[1]	**Age 12-14**, N = 11,426[1]	**Age 14-16**, N = 11,449[1]	**Age 16-18**, N = 11,265[1]
$\geq$ 6-month gap	1,610 (16%)	2,511 (23%)	2,663 (24%)	2,745 (24%)	2,887 (25%)	3,115 (28%)
$\geq$ 12-month gap	135 (1.4%)	430 (3.9%)	542 (4.8%)	556 (4.9%)	595 (5.2%)	631 (5.6%)

[1] n (%)

Note: individuals may contribute to more than one age category
