# Supplementary material for: Predictors of frequency of CF care in the US Cystic Fibrosis Foundation Patient Registry
Source: PLoS One. 2024 Dec 3;19(12):e0313510. doi: 10.1371/journal.pone.0313510 (PMC11614261; doi:10.1371/journal.pone.0313510)
Supplement: S6 Table — Confounding adjustment sets for the association between each predictor of interest and the study outcome, between visit interval. (PDF) [file pone.0313510.s008.pdf]

S6 Table. Confounding adjustment. Confounding adjustment sets for the association between each predictor of interest and the study outcome, between visit interval.

Model	Predictor of interest	Confounding adjustment
Sociodemographic factors		
**Model 1**	Age (linear and spline)	No confounding
**Model 2**	Sex	No confounding
**Model 3**	Race/Ethnicity	No confounding
**Model 4**	Rurality	No confounding
**Model 5**	Insurance	Age, non-white, education, income
**Model 6**	Insurance/Race	Age, education, income
**Model 7**	Education	Age, non-white
**Model 8**	Income	Sex, non-white, education, rurality
Disease-related factors		
**Model 9**	Genotype	Non-white
**Model 10**	Pulmonary impairment	Age, sex, genotype, underweight, chronic infections
**Model 11**	Underweight	Age, sex, income, genotype, CFRD, chronic infections
**Model 12**	CFRD	Age, sex, genotype
**Model 13**	Chronic infections (individual infection types)	Age, sex, CFRD
**Model 14**	Chronic infections (number of concurrent infections)	Age, sex, CFRD
**Model 15**	CF-related complications	Age, sex, insurance, genotype
