# Supplementary material for: Predictors of frequency of CF care in the US Cystic Fibrosis Foundation Patient Registry
Source: PLoS One. 2024 Dec 3;19(12):e0313510. doi: 10.1371/journal.pone.0313510 (PMC11614261; doi:10.1371/journal.pone.0313510)
Supplement: S7 Table — Sensitivity analyses evaluating multivariable regression results from the complete case analysis, after removing the between visit interval < 30 days exclusion criterion, and in a subset of people who are not experiencing CF-related complications (severe lung impairment defined as FEV1PP ≤ 40, underweight BMI, CF-related diabetes, and chronic infections). (PDF) [file pone.0313510.s009.pdf]

S7 Table. **Sensitivity analyses.** Sensitivity analyses evaluating multivariable regression results from the complete case analysis (exclusion of encounters missing any of our variables of interest), after removing the between visit interval < 30 days exclusion criterion, and in a subset of people who are not experiencing CF-related complications (severe lung impairment defined as FEV1PP ≤ 40, underweight BMI, CF-related diabetes, and chronic infections).

	**Primary Analysis**<br>pwCF = 28,588<br>encounters = 859,568			**Sensitivity Analysis: Complete Case**<br>pwCF = 14,764<br>encounters = 167,274			**Sensitivity Analysis: Without BVI < 30 day exclusion**<br>pwCF = 25,593<br>encounters = 1,325,092			**Sensitivity Analysis: No CF-related complications**<br>pwCF = 16,949<br>encounters = 186,578		
	**% Δ in BVI[1]**	**95% CI**	***P value***	**% Δ in BVI[1]**	**95% CI**	***P value***	**% Δ in BVI[1]**	**95% CI**	***P value***	**% Δ in BVI[1]**	**95% CI**	***P value***
Sociodemographic factors												
**Age at Encounter (decades, linear) [2,3]**	-0.6	-0.8, -0.3	<0.001	1.0	0.5, 1.6	<0.001	-14.4	-14.9, -14.0	<0.001	10.1	9.4, 10.8	<0.001
**Sex [3]**												
Male (female referent)	9.8	9.1, 10.5	<0.001	8.8	7.7, 9.9	<0.001	16.4	15.1, 17.8	<0.001	6.4	5.2, 7.7	<0.001
**Race/Ethnicity [3]**												
White and non-Hispanic (referent)												
Non-white or Hispanic	3.6	2.2, 5.0	<0.001	3.4	1.1, 5.7	0.003	1.7	-0.8, 4.2	0.2	4.5	2.2, 6.9	<0.001
**Rurality [3]**												
Urban (referent)												
Large rural	1.7	0.9, 2.6	<0.001	1.6	0.1, 3.2	0.032	2.7	1.1, 4.2	<0.001	0.9	-0.8, 2.6	0.3
Small rural	4.0	2.8, 5.1	<0.001	4.1	2.0, 6.2	<0.001	6.6	4.5, 8.7	<0.001	4.8	2.5, 7.2	<0.001
Isolated	1.2	-0.1, 2.5	0.063	-0.4	-2.7, 2.0	0.8	1.5	-0.7, 3.8	0.2	1.5	-1.2, 4.2	0.3
Unknown	8.4	6.4, 10.4	<0.001	-	-	-	10.3	6.7, 14.1	<0.001	8.2	4.3, 12.3	<0.001
**Insurance Coverage [4]**												
Private (referent)												
Medicare/Medicaid/ State Program/IHS	-3.4	-3.8, -3.0	<0.001	-3.9	-4.8, -3.0	<0.001	-5.6	-6.2, -4.9	<0.001	-0.5	-1.4, 0.5	0.3
Other Insurance	3.5	1.8, 5.1	<0.001	3.4	-0.8, 7.8	0.11	0.0	-2.9, 3.0	>0.9	1.3	-2.0, 4.7	0.5
No insurance	19.4	17.6, 21.2	<0.001	10.9	7.2, 14.8	<0.001	14.6	11.6, 17.7	<0.001	23.4	19.3, 27.6	<0.001
Unknown Insurance Status	13.0	11.8, 14.2	<0.001	-	-	-	3.8	1.9, 5.8	<0.001	18.3	15.5, 21.1	<0.001
**Education [5]**												
Less than high school education (referent)												

	**Primary Analysis**<br>pwCF = 28,588<br>encounters = 859,568			**Sensitivity Analysis: Complete Case**<br>pwCF = 14,764<br>encounters = 167,274			**Sensitivity Analysis: Without BVI < 30 day exclusion**<br>pwCF = 25,593<br>encounters = 1,325,092			**Sensitivity Analysis: No CF-related complications**<br>pwCF = 16,949<br>encounters = 186,578		
High School diploma or equivalent	0.3	-2.6, 3.2	0.9	2.6	-2.7, 8.3	0.3	3.7	-1.9, 9.5	0.2	-0.2	-4.7, 4.4	>0.9
Some College	-0.4	-3.3, 2.5	0.8	3.3	-2.0, 8.9	0.2	6.0	0.4, 11.9	0.035	-3.4	-7.7, 1.0	0.13
College Graduate	1.5	-1.3, 4.4	0.3	5.5	0.2, 11.1	0.042	16.7	10.7, 23.1	<0.001	-4.2	-8.3, 0.1	0.055
Masters/Doctoral level degree	1.9	-0.9, 4.9	0.2	6.6	1.2, 12.4	0.016	21.1	14.8, 27.8	<0.001	-7.9	-11.9, -3.7	<0.001
Missing	3.3	-0.1, 6.7	0.057	-	-	-	9.8	3.0, 17.1	0.004	-0.8	-5.6, 4.1	0.7
**Income** [6]												
<$40,000 (referent)												
$40,000 to $90,000	0.5	-0.2, 1.2	0.14	0.8	-0.1, 1.7	0.065	3.9	2.6, 5.1	<0.001	-0.1	-1.6, 1.4	0.9
>$90,000	1.1	0.3, 1.9	0.010	2.3	1.2, 3.4	<0.001	4.3	2.9, 5.8	<0.001	-0.7	-2.3, 1.0	0.4
Missing	3.1	2.2, 4.0	<0.001	-	-	-	3.5	1.9, 5.1	<0.001	5.3	3.5, 7.1	<0.001
Disease-related factors												
**Genotype** [7]												
F508del Heterozygote (referent)												
F508del Homozygote	-4.6	-5.2, -3.9	<0.001	-4.4	-5.4, -3.3	<0.001	-7.7	-8.8, -6.5	<0.001	-5.1	-6.3, -3.8	<0.001
Other/Unknown Mutation	4.5	3.5, 5.6	<0.001	5.3	3.5, 7.1	<0.001	6.7	4.7, 8.7	<0.001	4.8	3.1, 6.6	<0.001
**Pulmonary Impairment** [8]												
Mild (FEV1PP ≥70%, referent)												
Moderate (FEV1PP 41-69%)	-14.6	-14.9, -14.3	<0.001	-14.6	-15.3, -13.9	<0.001	-40.2	-40.6, -39.9	<0.001	-	-	-
Severe (FEV1PP ≤ 40%)	-27.6	-28.0, -27.2	<0.001	-27.8	-28.7, -26.8	<0.001	-60.7	-61.0, -60.3	<0.001	-	-	-
**Underweight BMI** [9]	-9.3	-9.8, -8.8	<0.001	-10.2	-11.3, -9.0	<0.001	-25.6	-26.2, -25.0	<0.001	-	-	-
**CF-related Diabetes** [10]	-16.2	-16.6, -15.8	<0.001	-15.2	-16.0, -14.4	<0.001	-27.8	-28.4, -27.2	<0.001	-	-	-
**Chronic Infections** [11]												
P. aeruginosa	-10.5	-10.9, -10.1	<0.001	-11.5	-12.4, -10.7	<0.001	-17.6	-18.2, -16.9	<0.001	-	-	-
MRSA	-10.4	-10.8, -10.0	<0.001	-10.2	-11.0, -9.4	<0.001	-18.8	-19.4, -18.1	<0.001	-	-	-
Burkholderia spp.	-10.2	-11.1, -9.3	<0.001	-8.5	-10.3, -6.7	<0.001	-19.2	-20.6, -17.8	<0.001	-	-	-
**Prior CF-related Complications** [12]												
Complications (referent)												
No complications	16.0	15.4, 16.5	<0.001	17.2	16.0, 18.4	<0.001	34.8	33.6, 35.9	<0.001	-	-	-

[1] Adjusted percent difference in between visit interval = $100(\exp(\beta) - 1)\%$

[2] Per 10 year increase in age, linear model without use of splines

[3] No confounding adjustment
[4] Insurance: Adjusted for age, non-white, education, income
[5] Education: Adjusted for age, non-white
[6] Income: Adjusted for sex, non-white, education, rurality
[7] Genotype: Adjusted for non-white
[8] Pulmonary impairment: Adjusted for age, sex, genotype, underweight, chronic infections
[9] Underweight: Adjusted for age, sex, income, genotype, CFRD, chronic infections
[10] CF-related diabetes: Adjusted for age, sex, genotype
[11] Chronic infections: Adjusted for age, sex, CFRD
[12] Prior CF-related Complications: Adjusted for age, sex, insurance, genotype
BVI = between-visit interval, MRSA = Methicillin-resistant *Staphylococcus aureus,* FEV1PP = Forced expiratory volume in one second, CFRD = cystic fibrosis-related diabetes
