# Supplementary material for: Predictors of frequency of CF care in the US Cystic Fibrosis Foundation Patient Registry
Source: PLoS One. 2024 Dec 3;19(12):e0313510. doi: 10.1371/journal.pone.0313510 (PMC11614261; doi:10.1371/journal.pone.0313510)
Supplement: S8 Table — Pediatric is defined as <18 and adults are ≥18 years of age. (PDF) [file pone.0313510.s010.pdf]

S8 Table. Sensitivity analysis evaluating multivariable results stratified by age group. Pediatric is defined as <18 and adults are ≥18 years of age.

	**Pediatric**<br>pwCF = 18,924<br>encounters = 433,615			**Adult**<br>pwCF = 19,096<br>encounters = 425,953		
	**% Δ in BVI**[1]	**95% CI**	***P value***	**% Δ in BVI**[1]	**95% CI**	***P value***
***Sociodemographic** factors*						
**Age at Encounter (decades, linear)** [2,3]	-6.8	-7.3, -6.3	<0.001	0.1	-0.3, 0.5	0.6
**Sex** [3]						
Male (female referent)	4.9	4.1, 5.7	<0.001	12.9	11.9, 13.9	<0.001
**Race/Ethnicity** [3]						
White and non-Hispanic (referent)						
Non-white or Hispanic	3.6	2.1, 5.1	<0.001	5.4	3.3, 7.5	<0.001
**Rurality** [3]						
Urban (referent)						
Large rural	2.5	1.4, 3.6	<0.001	1.5	0.3, 2.8	0.016
Small rural	4.1	2.7, 5.5	<0.001	4.3	2.6, 6.1	<0.001
Isolated	1.6	0.1, 3.2	0.037	1.2	-0.7, 3.2	0.2
Unknown	6.9	4.4, 9.6	<0.001	9.6	6.7, 12.6	<0.001
**Insurance Coverage**[4]						
Private (referent)						
Medicare/Medicaid/State Program/IHS	-0.7	-1.2, -0.1	0.013	-6.0	-6.6, -5.4	<0.001
Other Insurance	1.1	-1.2, 3.4	0.4	5.3	2.8, 7.8	<0.001
No insurance	14.4	11.3, 17.5	<0.001	20.4	18.1, 22.7	<0.001
Unknown Insurance Status	10.6	8.9, 12.3	<0.001	15.0	13.2, 16.8	<0.001
**Insurance/Race** [5]						
Private, White versus Non-White	-3.4	-5.1, -1.7	<0.001	-5.1	-7.4, -2.8	<0.001
Public, White versus Non-White	-3.6	-5.2, -2.0	<0.001	-7.3	-9.4, -5.0	<0.001

Other, White versus Non-White	-3.1	-6.7, 0.7	0.11	-5.6	-9.7, -1.3	0.012
**Education** [6]						
Less than high school education (referent)						
High School diploma or equivalent	1.7	-1.1, 4.6	0.2	-3.4	-8.2, 1.7	0.2
Some College	3.0	0.2, 5.8	0.037	-6.3	-10.9, -1.4	0.012
College Graduate	4.2	1.5, 7.0	0.002	-3.8	-8.5, 1.1	0.13
Masters/Doctoral level degree	4.3	1.5, 7.2	0.003	-3.2	-7.9, 1.8	0.2
Missing	4.3	1.2, 7.5	0.006	19.5	10.2, 29.5	<0.001
**Income** [7]						
<$40,000 (referent)						
$40,000 to $90,000	0.2	-0.6, 1.1	0.6	1.2	0.1, 2.2	0.026
>$90,000	0.6	-0.4, 1.7	0.2	1.6	0.4, 2.8	0.007
Missing	2.0	0.9, 3.1	<0.001	3.0	1.8, 4.2	<0.001
Disease-related factors						
**Genotype** [8]						
F508del Heterozygote (referent)						
F508del Homozygote	-3.0	-3.8, -2.2	<0.001	-5.3	-6.2, -4.3	<0.001
Other or Unknown Mutation	4.4	3.2, 5.7	<0.001	4.8	3.4, 6.2	<0.001
**Pulmonary Impairment** [9]						
Mild (FEV1PP ≥70%, referent)						
Moderate (FEV1PP 41-69%)	-14.4	-14.8, -14.0	<0.001	-15.3	-15.7, -14.8	<0.001
Severe (FEV1PP ≤ 40%)	-23.8	-24.6, -22.9	<0.001	-29.0	-29.6, -28.4	<0.001
**Underweight BMI** [10]	-9.1	-9.8, -8.4	<0.001	-9.9	-10.5, -9.2	<0.001
**CF-related Diabetes** [11]	-13.5	-14.1, -13.0	<0.001	-18.7	-19.3, -18.2	<0.001
**Chronic Infections** [12]						
P. aeruginosa	-9.2	-9.6, -8.7	<0.001	-14.0	-14.6, -13.3	<0.001
MRSA	-8.4	-8.9, -7.9	<0.001	-12.4	-13.1, -11.8	<0.001
Burkholderia spp.	-10.1	-11.4, -8.9	<0.001	-11.3	-12.6, -10.0	<0.001
**Prior CF-related Complications** [13]						
Complications (referent)						
No complications	13.1	12.6, 13.7	<0.001	28.3	27.1, 29.5	<0.001

[1] Adjusted percent difference in between visit interval = 100(exp(β) − 1)%
[2] Per 10 year increase in age, linear model without use of splines
[3] No confounding adjustment
[4] Insurance: Adjusted for age, non-white, education, income

[5] Insurance/Race: Adjusted for age, education, income
[6] Education: Adjusted for age, non-white
[7] Income: Adjusted for sex, non-white, education, rurality
[8] Genotype: Adjusted for non-white
[9] Pulmonary impairment: Adjusted for age, sex, genotype, underweight, chronic infections
[10] Underweight: Adjusted for age, sex, income, genotype, CFRD, chronic infections
[11] CF-related diabetes: Adjusted for age, sex, genotype
[12] Chronic infections: Adjusted for age, sex, CFRD
[13] Prior CF-related Complications: Adjusted for age, sex, insurance, genotype
BVI = between-visit interval, MRSA = Methicillin-resistant *Staphylococcus aureus,* FEV1PP = Forced expiratory volume in one second, CFRD = cystic fibrosis-related diabetes
