# Supplementary material for: Predictors of frequency of CF care in the US Cystic Fibrosis Foundation Patient Registry
Source: PLoS One. 2024 Dec 3;19(12):e0313510. doi: 10.1371/journal.pone.0313510 (PMC11614261; doi:10.1371/journal.pone.0313510)
Supplement: S1 File — (PDF) [file pone.0313510.s011.pdf]

S1 File. Model Specifications, Example Code, and Description of Sensitivity Analyses.

Model Specification.

Methods were similar to those used by Sears, et al. [1]. We used a linear mixed effect model. Suppose there are N patients in the USCFFPR indexed by $i = 1, 2, \ldots, N$. Let $FEV1PP_{ij}$ represent lung function measured for the ith patient at the jth time, represented as age_{ij} (corresponding to patient age, in years), $j = 1, \ldots, n_i$. The longitudinal model of the time between encounters consists of a linear mixed model with random effects:

$$\textit{Time between encounters}_{ij} = f(age_{ij}) + m_i(age_{ij}) + W_{1i}(age_{ij}) + \varepsilon_{ij},$$

where the population-level mean response $f(age_{ij})$ is modeled as a continuous function of time, which combines the time-related fixed effects terms and regression splines; $m_j(age_{ij})=x'_i(age_{ij})\beta$ refers to the subject-level fixed effects. The vector $\mathbf{x}_i(age_{ij})$ represents static and time-varying covariates defined above, and the vector β contains their corresponding regression coefficients. The expression $W_i(age_{ij})=z'_i(age_{ij})\mathbf{U_i}$ incorporates random effects that describe how subject-specific true time between encounters deviate from their expected behavior, where the vector $\mathbf{U}_i$ corresponds to subject-specific random slopes and intercepts, $\mathbf{U_i}=(U_{0i}, U_i)'$. We used the linear form, $W_i(age_{ij}) = U_{0i} + U_i age_{ij}$, corresponding to $\mathbf{z}_i(age_{ij}) = (1, age_{ij})'$; this specification allows individuals to have varying baseline time between encounters and different lengths of time between encounters across age. Lastly, the term $\varepsilon_{ij} \sim N(0,\sigma^2_\varepsilon)$ denotes zero-mean Gaussian measurement error. We used a nonlinear semiparametric representation of $f(age_{ij})$ through the inclusion of natural cubic splines in order to provide smooth, nonlinear estimates of the longitudinal course of the time between encounters. We selected the number and location of knots (κ_l, $l = 1, \ldots, L$) for the corresponding spline basis functions using a quantile-based approach. The six knots used across the [6, 60] age (in years) interval were κ = (6.0, 10.5, 14.2, 18.0, 22.9, 30.9, 60.0).

Example Code. Example code provided for models testing the association between the interval between visits and insurance coverage (Model 5) and this association's interaction with age.

Model 5: BVI ~ Insurance type, adjusted for age, non-white race/ethnicity, highest family education, and family income

```
Model5 <- nlme::lme(log(BVI) ~ 1 + insurance_type + ns(encounterage, df = 6) + non_white + highest_ed + family_income, random = ~ 1 | CF_Clinic_ID / Patient_ID, data = full_cohort, method='ML', control = lmeControl(opt = "optim"))
```

Model 5a: BVI ~ Insurance type, adjusted for age, non-white race/ethnicity, highest family education, and family income with an interaction term between insurance type and age

```
Model5a <- nlme::lme(log(BVI) ~ 1 + insurance_type*ns(encounterage, df = 6) + non_white + highest_ed + family_income, random = ~ 1 | CF_Clinic_ID / Patient_ID, data = full_cohort, method='ML', control = lmeControl(opt = "optim"))
```

1. Sears EH, Jr., Hinton AC, Lopez-Pintado S, Lary CW, Zuckerman JB. Gaps in Cystic Fibrosis Care Are Associated with Reduced Lung Function in the U.S. Cystic Fibrosis Foundation Patient Registry. Ann Am Thorac Soc. 2023;20(9):1250-7.

Description of Sensitivity Analyses: Sensitivity analyses were conducted to evaluate the effect of five analytic decisions. First, the multivariable models were run on a cohort that excluded all encounters missing data for any of the predictors of interest (complete case analysis). Second, we ran the analyses including encounters closer together than 30 days. Third, we conducted the same analyses in a subgroup of encounters where patients did not have CF-related complications (severe lung impairment defined as FEV1PP ≤ 40, underweight BMI, CFRD, and chronic infections) at the time of their encounter. Fourth, the models were run separately in pediatric (age ≤18) and adult (age >18) pwCF to evaluate whether predictors of BVI differed in these populations. Finally, education was dichotomized into those with a college degree and those without (at the family-level).
